# Individualized medication based on pharmacogenomics and treatment progress in children with IgAV nephritis

**DOI:** 10.3389/fphar.2022.956397

**Published:** 2022-07-22

**Authors:** Xuerong Yang, Qi Li, Yuanyuan He, Yulian Zhu, Rou Yang, Xiaoshi Zhu, Xi Zheng, Wei Xiong, Yong Yang

**Affiliations:** ^1^ Department of Pharmacy, Sichuan Academy of Medical Sciences & Sichuan Provincial People’s Hospital, School of Medicine, University of Electronic Science and Technology of China, Chengdu, China; ^2^ Personalized Drug Therapy Key Laboratory of Sichuan Province, School of Medicine, University of Electronic Science and Technology of China, Chengdu, China; ^3^ Department of Pharmacy, Ziyang People’s Hospital, Ziyang, China; ^4^ Department of Pediatrics, Sichuan Academy of Medical Sciences & Sichuan Provincial People’s Hospital, Chengdu, China; ^5^ Department of Hepatobiliary Surgery, Sichuan Academy of Medical Sciences & Sichuan Provincial People’s Hospital, Chengdu, China

**Keywords:** IgA vasculitis nephritis, Henoch-Schönlein purpura nephritis, treatment, immunosuppressants, personalized medicine, pharmacogenomics, gene polymorphism, paediatric

## Abstract

Immunoglobulin A vasculitis (IgAV) nephritis, also known as Henoch-Schönlein purpura nephritis (HSPN), is a condition in which small blood vessel inflammation and perivascular IgA deposition in the kidney caused by neutrophil activation, which more often leads to chronic kidney disease and accounts for 1%–2% of children with end-stage renal disease (ESRD). The treatment principles recommended by the current management guidelines include general drug treatment, support measures and prevention of sequelae, among which the therapeutic drugs include corticosteroids, immunosuppressive agents and angiotensin system inhibitors. However, the concentration range of immunosuppressive therapy is narrow and the individualized difference is large, and the use of corticosteroids does not seem to improve the persistent nephropathy and prognosis of children with IgAV. Therefore, individualized maintenance treatment of the disease and stable renal prognosis are still difficult problems. Genetic information helps to predict drug response in advance. It has been proved that most gene polymorphisms of cytochrome oxidase P450 and drug transporter can affect drug efficacy and adverse reactions (ADR). Drug therapy based on genetics and pharmacogenomics is beneficial to providing safer and more effective treatment for children. Based on the pathogenesis of IgAV, this paper summarizes the current therapeutic drugs, explores potential therapeutic drugs, and focuses on the therapeutic significance of corticosteroids and immunosuppressants in children with IgAV nephritis at the level of pharmacogenomics. In addition, the individualized application of corticosteroids and immunosuppressants in children with different genotypes was analyzed, in order to provide a more comprehensive reference for the individualized treatment of IgAV nephritis in children.

## 1 Introduction

Immunoglobulin A vasculitis (IgAV), previously known as Henoch-Schönlein purpura, is the most common systemic vessel vasculitis in children. It’s a kind of immune sediment dominated by IgA and usually self-limiting, which often affects the skin (which can touch purpura), gastrointestinal tract, lungs, joints, nervous system, urinary system, and renal (Nicoara and Twombley, 2019). The incidence of IgAV is about 3–27 cases in every 100,000 children (Piram et al., 2017), and it can appear at any age, with the highest incidence being about four to 6 years old and 90% of cases happening under 10 years old (Oni and Sampath, 2019). Males are slightly dominant (the ratio of men to women is 1.5: 1), and the incidence tends to decrease with age (Gardner-Medwin et al., 2002). The prognosis for IgAV in children is generally reasonable. However, serious complications can still occur, including renal involvement, with IgAV nephritis progressing in the acute phase of IgAV involving the kidneys, which can occur in 20%–80% of IgAV cases (Ozen et al., 2019). Most children with IgAV nephritis are slightly ill, exhibiting only hematuria or light proteinuria, and likely to recover independently. However, the long-term prognosis of patients with IgAV is determined by the severity of renal involvement; a small percentage of patients develop nephrotic syndrome or renal impairment that may progress to renal failure or end-stage renal disease. Therefore, it is very crucial for children and families to choose the most appropriate therapeutic drugs based on a clear understanding of the pathogenesis and clinical manifestations of IgAV nephritis. The management of IgAV is still highly controversial, with few international management guidelines and no consensus between regions.

Currently, in addition to the widely recognized renin-angiotensin system (RAS) inhibitors, drugs such as corticosteroids (CS) and various immunosuppressive agents are often recommended for IgAV nephritis in the presence of proteinuria to stifle renal damage (Ozen et al., 2019). However, the therapeutic range of immunosuppressive drugs is very narrow, which suggests that immunosuppression can easily fall between efficacy and toxicity (Casati et al., 2017). Since the dosage and monitoring target concentrations of drugs used to treat IgAV nephritis in children are inferred from experience with adults, these drugs have not been sufficiently validated for use in children and are prone to imprecise dosages, which can adversely affect growth and development in children.

Genetics is and mayone of the most common factors associated with drug response, and having genetic information about patients can help predict drug response in advance. Due to the narrow concentration range of immunosuppressive drug therapy, pharmacogenomics is expected to be one of the most promising tools in the field of immunosuppressive drug therapy. It may help select appropriate drugs for children with IgAV nephritis and individualize the dose (Collins and Varmus, 2015). In this review, we compare the current major international guidelines, summarize the current therapeutic use based on the analysis of the mechanisms of IgAV occurrence and explore potential therapeutic agents. We focus on the impact of genetic polymorphisms of cytochrome oxidase P450, drug transporters, and pharmacodynamic drug targets on hormones and immunosuppressants, and analyze the individualized application of hormones and immunosuppressants in children with different genotypes in order to achieve precise drug use in children with IgAV nephritis and provide a more comprehensive reference for the individualized treatment of IgAV nephritis in children. This review is dedicated to supplying some information for future individualized treatment based on pharmacogenomics and pharmacogenetics.

## 2 Immunoglobulin A vasculitis nephritis

### 2.1 Pathogenesis of immunoglobulin A vasculitis nephritis

IgAV nephritis is a typical acute glomerular inflammatory disease caused by autolytic vasculitis. It is due to mesangial proliferative changes caused by aberrant glycosylation of IgA1 sedimentation in the glomerulus. At present, the precise mechanism for this situation is unclear. Kiryluk et al. (2017) proposed that crucial gene abnormalities may cause it in the glycosylation pathway. Such aberrant glycosylation leads to the exposure of residues in the IgA hinge region, which constitutes antigens and triggers humoral autoimmune responses, and its cycle immunity compound and immune deposition include IgA1. Aberrant glycosylation may be associated with serum galactose deficiency variation, which is seldom seen in normal circulating IgA1 but is more frequently seen in IgAV people. Specifically, transferrin receptors preferentially bind to galactose deficient IgA1 in the renal and express it on its mesangial cells, forming deposits through enhanced cell multiplication, cytokine release, completement activation, induction of Pro cell-extracellular matrices (ECM), ultimately leading to local renal tissue inflammation (Oni and Sampath, 2019). Studies have shown that the primary circulating sediment is a large (>19S) circulating IgA1-o-sugar-containing immune complex (IgA-IC) (Nicoara and Twombley, 2019). It is unclear whether these circulating complexes and immune deposits are temporary phenomena in the acute phase or even present in the quiescent phase of the disease (Nicoara and Twombley, 2019; Oni and Sampath, 2019).

In addition to IgA1 deposition, IgAV nephritis has also been found to be correlated with immune factors, including immune complexes, and its incidence is positively correlated with plasma IgG and IgE levels and type 1 allergic reactions (Nicoara and Twombley, 2019).

### 2.2 Clinical manifestations of immunoglobulin A vasculitis nephritis

IgAV is a vasculitis of small vessel leukocyte fragmentation; compared with the clinical manifestations of adult IgAV nephritis, children’s IgAV nephritis clinical manifestations are different (Oni and Sampath, 2019). In 95% of patients, a skin rash will have a symmetrical red spot or palpable purpura rash; most start in the legs and hips and can extend to the arms and less common trunk (Oni and Sampath, 2019). Facial involvement is sporadic. With the exception of skin symptoms, the disease is characterized by typical triad symptoms, namely, involving the skeletal, muscle and kidney systems, rarely involving the respiratory system, and very rarely involving the nervous system. In the brachychronic phase, patients with musculoskeletal involvement up to 70%–90%, manifested as joint pain or arthritis. The incidence of arthritis is relatively low, about 61%–64%; the most common involvement is lower limb joints, which are likely to occur in the same area as rashes, but joint symptoms are usually transient and do not cause long-term abnormalities (Oni and Sampath, 2019). As many as 72% of patients develop gastrointestinal symptoms, usually gastrointestinal colic, which may precede skin manifestations for several days or even a week. In severe cases, acute gastrointestinal bleeding, such as melena, or severe and life-threatening vomiting may be developed. However, asymptomatic fecal occults are common (Oni and Sampath, 2019). Renal involvement, called IgAV nephritis, is the most severe manifestation of IgAV. About 20%–80% of children may be involved in the renal, which is usually asymptomatic and requires activity screening.

IgAV nephritis can be manifested as hematuria with or without albumin urine, nephropathy and/or idiopathic nephrotic syndrome under the microscope (and/or visible to the naked eye), and/or purpura, colic, bloody stools, edema, and joint pain. In general, IgAV nephritis is somewhat self-limited and may recover without treatment. Children with mild IgAV nephritis have a good prognosis but may develop permanent renal damage and scarring if not diagnosed and treated early (Chan et al., 2016). IgAV nephritis in children is generally restricted to abnormal urine, but there are no clinical symptoms. At the same time, the blood pressure and renal function of children are standard, making it difficult to monitor and manage IgAV nephritis in children. Indeed, the long-run risk of lasting renal dysfunction in patients with minor urinary abnormalities is low but significantly higher in children with nephrotic and/or nephrotic syndrome (Narchi, 2005). The risk of developing chronic kidney disease in children with mild renal involvement is only between 5 and 20 percent (Bogdanovic, 2009). Therefore, it is important for all children suspected of IgAV to be actively examined for renal involvement during diagnosis. At least 6–12 months of monitoring and follow-up are required, even though the original blood pressure and urine test are not abnormal.

Renal involvement needs to be evaluated by measuring blood pressure, morning urine test, and glomerular filtration rate (GFR) to evaluate the comprehensive renal function. Urine analysis (morning urine samples) should confirm hematuria, proteinuria, and/or proteinuria quantification and urine protein: urine creatinine ratio (UP: UC ratio) (Foster and Brogan, 2018). The calculation method of GFR in children is different from that in adults. The simplest and most commonly used method is the improved Schwartz formula to estimate the renal function of children aged 1–17. The accuracy of calculation and diagnostic accuracy of the formula has been verified, that is, eGFR (ml/min/1.73 m^2^) = 36.2 × height (cm)/serum creatinine (μmol/L). However, the formula is derived from GFR in children 15–75 ml/min/1.73 m^2^, and it may be invalid if GFR exceeds this range (Bhowmick et al., 2022). The classical Schwartz formula was used to estimate renal function in 0–12 months infants. The GFR of low-birth-weight infants with a birth weight less than 2.5 kg was 29.1 × body length (cm)/serum creatinine (μmol/L), and the GFR of full-term infants was 39.7 × body length (cm)/serum creatinine (μmol/L). At the same time, renal biopsy should be performed for histological classification based on GFR impairment or severe or persistent proteinuria to more accurately assess the severity of IgAV nephritis.

## 3 Genetic susceptibility to immunoglobulin A vasculitis nephritis

Recent studies have confirmed that individuals with IgAV nephritis have a strong genetic susceptibility. It is correlated with human leukocyte antigen (HLA), especially in its type II region (Gonzalez-Gay et al., 2018). Currently, genetic susceptibility to IgAV nephritis is mainly studied in The European population. A study of 349 patients with IgAV showed that Caucasian people have associated with HLA type I alleles, especially HLA-B*41:02. This study is inconsistent with the previous research (Lopez-Mejias et al., 2015b), which was relevant to the HLA-DRB1*01 allele (Lopez-Mejias et al., 2015a). The claim that HLA Class II regions constitute a significant susceptibility locus for IgAV has been supported by the largest ever genetic study of IgAV patients of European descent, which confirmed that the mapping of a link disequilibrium region between HLA-dqa1 and HLA-dqb1 to the intergenic region (IGR) of HLA class II was closely related to the susceptibility of disease. The values of HLA-DRB1*13 and HLA-DRB1*11 were very high; weak correlation with HLA class I regions and latent signals other than HLA were found (Lopez-Mejias et al., 2017).

Other studies have also shown that susceptibility to IgAV nephritis is associated with genetic polymorphisms outside the HLA region, containing genetic polymorphisms that regulate vascular homeostasis, new angiogenesis, and gene polymorphisms that encode aberrant glycosylation of IgA1(Lopez-Mejias et al., 2018). It was also confirmed that T cells, pro-inflammatory cytokines, or homocysteine metabolism might be related to the susceptibility to IgAV and the universality of IgAV (Lopez-Mejias et al., 2018). Another study evaluated the relevance of cytokines and immune globulin with glomerulonephritis in IgAV children and observed IgA1 levels in serum galactose deficiency and IgA, IgG, IgM, IL-6, IL-8, and IL-10 complex levels in urine were predictors of glomerulonephritis in IgAV children (Berthelot et al., 2018).

## 4 Drug therapy for immunoglobulin A vasculitis nephritis in children

### 4.1 Renin-angiotensin system blockers

It has been shown that blocking the RAS is beneficial to renal function (Selvaskandan et al., 2019). Regardless of whether prednisolone or other immunosuppressants are used in treatment, the panel recommended that angiotensin-converting enzyme inhibitors (ACEI) or angiotensin II receptor blockers (ARBs) for IgAV in children with continuous proteinuria (>3 months) and renal involvement to prevent or restrict subsequent glomerular injury (Coppo et al., 2007). Also, the Kidney Disease Improving Global Outcomes (KDIGO) group approved RAS blockers for treating persistent proteinuria, and the KDIGO guidelines (Rovin et al., 2021) recommend that children with persistent proteinuria with IgAV nephritis (defined as > 0.5–1 g/d/1.73 m^2^) should be treated with ACEI or ARBs.

The efficacy of RAS blockers on IgAV cannot be clearly demonstrated. Still, clinical studies suggest that RAS blocker use has a favorable impact on the nephritic outcomes of patients with IgAV. In a recent study (Nagai et al., 2022), proteinuria was resolved in cases of moderate-to-severe IgAV nephritis with RAS inhibitors as the initial treatment regimen, regardless of relapse. Irrespective of the status of pathological findings in children diagnosed with IgAV, RAS inhibitor therapy at an early stage has shown excellent results and beneficial effects on renal outcomes (Kurt-Sukur et al., 2021; Nagai et al., 2022). This suggests that timely assessment of patients’ clinical and pathologic outcomes prior to therapeutic intervention and early introduction of RAS inhibitors as a treatment strategy for IgAV nephritis is exceptionally beneficial. Some guidelines recommend that in the absence of crescentic nephritis (>50% of crescents) with worsening renal function and nephrotic syndrome, treatment with only ACEI or ARBs alone may be used initially for 3–6 months (Radhakrishnan and Cattran, 2012). Other experts, however, believe this approach may leave acute and potentially aggressive glomerulonephritis undetermined or its immunosuppressive management deferred for months, leading to undertreatment (Davin and Coppo, 2013). Relapse is more likely with RAS blockers alone, especially when the patient has global/segmental sclerosis or tubular atrophy/interstitial fibrosis (Nagai et al., 2022). Therefore, RAS blockers combined with other therapies such as CS and immunosuppressive agents can be considered in more complex cases.

### 4.2 Corticosteroids

The potent anti-inflammatory effect of CS can inhibit the rapid deterioration of renal pathology in the IgAV patients’ population to a certain extent, especially in diminishing urinary protein and postponing the renal function decline. In patients’ serum, CS can reduce the aberrant o-glycosylation of IgA1, thereby reducing the latter’s deposition in the glomerular capillary wall and mesangial. The guideline of 2012 KDIGO recommended that patients with persistent proteinuria be treated with CS for 6 months after using ACEI or ARBs (KDIGO, 2012). In the latest guidelines of KDIGO in 2021, oral prednisone/prednisone or pulsed intravenous (IV) methylprednisolone (MP) were recommended for the treatment of patients with mild and moderate IgAV nephritis (Rovin et al., 2021). Recommendations for CS use in China are basically consistent with the 2012 KDIGO. For children with severe clinical symptoms, diffuse renal pathological changes, or with > 50% crescent formation, methylprednisolone can be used for shock therapy in addition to oral CS (see Table 1 for the severity of the condition) (Subspecialty Group of Renal Diseases and Chinese Medical Association, 2017). The SHARE (Single Hub and Access Point for Paediatric Rheumatology in Europe) initiative issued first the international evidence-based recommendation on treating IgAV in children, and CS application in children with kidney injury was made more evident (Ozen et al., 2019). Oral or IV CS runs through severe, moderate, and even mild IgAV nephritis treatment, with a 10–30 mg/kg recommended dosage of pulsed MP. Patients can use it for 3 days, up to 1 g daily. Subsequently, in the latest evidence-based consensus in Egypt, oral prednisolone or pulsed MP is recommended for mild, moderate, and severe nephritis (see Table 1: Comparison of guidelines for the severity of the condition) (Abu-Zaid et al., 2021). The publication of these guidelines and consensus has given the international community a clearer understanding of the treatment of IgAV in children. However, there are still many problems here.

**TABLE 1:** Comparison of guidelines.

Guideline	Severity of IgAV nephritis and definition	Treatment recommendations
Evidence-based guideline for diagnosis and treatment of Henoch-Schönlein purpura nephritis (2016) Subspecialty Group of Renal Diseases and Chinese Medical Association (2017)	Isolated hematuria or pathological grade I (slight glomerular abnormality).	Only complementary treatment for HSP.
	Isolated microalbuminuria or combined with microscopic hematuria or pathological grade Ⅱ a (focal segmental simple mesangial hyperplasia).	ACEI or ARBs can be routinely used.
	Non-nephropathic proteinuria or pathological grade II b (diffuse simple mesangial hyperplasia), grade III a (focal segmental mesangial hyperplasia, with < 50% glomerular crescent formation/segmental lesions).	CS for 6 months.
	Nephropathy level proteinuria, nephrotic syndrome, acute nephritis syndrome or pathological grade III b (diffuse mesangial hyperplasia, with < 50% crescent formation/segmental glomerular lesions), grade IV (lesions the same as grade III, 50%–75% glomeruli with the above lesions).	Available regimens:
		1. CS combined with CYC pulse therapy: prednisone 1.5–2 mg/kg/d, oral administration for 4 weeks, and then oral administration every other day for 4 weeks, followed by IV pulse therapy with CYC based on CS. CYC accumulation ≤ 168 mg/kg.
		2. CS combined with CyA: CyA was taken orally four times at 6 mg/kg/d every 12 h. The serum concentration was measured 1–2 weeks after administration, and the trough concentration was maintained at 100–200 μg/L. The induction period was 3 μg/L for 6 months.
		3. CS combined with MMF: MMF 20–30 mg/kg/d was taken orally twice and gradually decreased after 3–6 months. The complete course of treatment was 12–24 months.
		4. CS combined with AZA: AZA 2 mg/kg/d, the general course of treatment was 8 months to 1 year.
	Acute nephritis or pathological grade V (lesions the same as grade III, > 75% of glomeruli with the above lesions), grade VI (mesangial proliferative glomerulonephritis).	A combination of 3–4 drugs:
		1. Prednisone + CYC (or other immunosuppressants) + heparin + dipyridamole were taken orally after 1–2 courses of MP pulse therapy.
		2. MP combined with urokinase shock therapy + oral prednisone + CYC + heparin + dipyridamole.
European consensus-based recommendations for diagnosis and treatment of immunoglobulin A vasculitis—the SHARE initiative Ozen et al. (2019)	Mild IgAV nephritis: normal GFR (>80 ml/min/1.73 m^2^) and mild proteinuria (UP: UC ratio < 100 mg/mmol) or moderate proteinuria (UP: UC ratio 100–250 mg/mmol).	1st line: oral prednisolone.
		2nd line: AZA, MMF, pulsed MP.
	UP: UC is tested in an early morning urine sample.	ACEI can be considered a preventive drug for (persistent) albuminuria.
	Moderate IgAV nephritis: < 50% crescents on renal biopsy and GFR damage (<80 ml/min/1.73 m^2^) or severe persistent proteinuria (>250 mg/mmol for 4 weeks).	1st line: oral prednisolone (1–2 mg/kg/d) or pulsed MP (10–30 mg/kg, up to 1 g/d for 3 days).
		2nd line: AZA, MMF, IV CYC.
		Oral CyA or CYC is not routinely indicated.
	Severe IgAV nephritis: > 50% crescents on renal biopsy and GFR damage or severe persistent proteinuria. (The definition is the same as the corresponding in Moderate IgAV nephritis.)	1st line: High-dose CS and IV CYC as induced therapy.
		2nd line: Low-dose CS + AZA/MMF as maintenance therapy.
Executive summary of the KDIGO 2021 guideline for the management of glomerular diseases Rovin et al. (2021)	Persistent proteinuria (>0.5–1 g/d/1.73 m^2^) for more than 3 months.	ACEI or ARBs (the combination of these two drugs is not recommended).
		Glucocorticoids are not recommended to prevent nephritis in patients with isolated extrarenal IgAV.
	Mild or Moderate nephritis	Oral prednisone/prednisolone or pulsed MP.
	Nephrotic syndrome or rapid deterioration of renal function	CYC + glucocorticoids (usually as pulsed MP).
Consensus evidence-based recommendations for treat-to-target management of immunoglobulin A vasculitis Abu-Zaid et al. (2021)	Mild nephritis: normal eGFR (>90 ml/min) and mild or moderate proteinuria, microscopic hematuria.	1st line: oral prednisolone.
		2nd line: AZA, MMF, pulsed MP (drug-resistant cases with no response).
	Moderate nephritis: < 50% crescents on renal biopsy and eGFR damage (60–89 ml/min/1.73 m^2^) or severe persistent proteinuria.	1st line: oral prednisolone and/or pulsed MP.
		2nd line: AZA, MMF, IV CYC.
	Severe nephritis: > 50% crescents on renal biopsy and eGFR damage (<60 ml/min) or severe persistent proteinuria.	1st line: IV CYC + pulsed MP and oral prednisolone for 6–9 months as induction therapy.
		2nd line: AZA/MMF + CS as maintenance therapy.
	Persistent proteinuria (UP: UC ratio > 250 mg/mmol for 4 weeks or UP: UC ratio > 100 mg/mmol for 3 months or UP: UC ratio > 50 mg/mmol for 6 months), drug-resistant cases, incurable or recurrent IgAV.	1. Children with persistent proteinuria (0.5–1.0 g/d) should be treated with ACEI or ARBs.
		2. After ACEI or ARBs tests, children with persistent proteinuria > 1 g/d and GFR > 50 ml/min/1.73 m^2^ should be treated with CS for 6 months (oral administration of 0.5 mg/kg prednisone or 0.8–1 mg/kg prednisone every other day for 2 months, followed by a monthly decrease of 0.2 mg/kg/d in the next 4 months).
		3. Plasmapheresis and IV immunoglobulin can be used in drug-resistant cases.
		4. Rituximab is recommended as replacement therapy for recurrent or incurable IgAV.

IgAV, IgA vasculitis; HSP, Henoch-Schönlein purpura; ACEI, angiotensin-converting enzyme inhibitors; ARBs, angiotensin II, receptor blockers; CS, corticosteroids; CYC, cyclophosphamide; IV, intravenous; CyA, cyclosporine A; MMF, mycophenolate mofetil; AZA, azathioprine; MP, methylprednisolone; GFR, glomerular filtration rate; UP, UC; urine protein, urine creatinine ratio; eGFR, estimated glomerular filtration rate.

Significantly, long-term CS administration can induce the inhibition of growth and development, osteoporosis, diabetes, adrenal inhibition, and Cushing syndrome (Caplan et al., 2017; Adami and Saag, 2019; Tan et al., 2019). More importantly, a considerable number of patients in the clinical use of CS will not respond or show increased disease, CS resistance phenomenon, which may be related to pharmacogenomics. Pharmacogenomics of CS has been extensively studied in many conditions. Related studies have shown that the key reasons for the individualized diversity of CS in humans are single nucleotide polymorphisms (SNPs) of nuclear receptor subfamily 3 group C member 1 (NR3C1), corticotropin-releasing hormone receptor 1 (CRHR1), glucocorticoid-induced transcript 1 gene (GLCC1), stress-induced phosphoprotein 1 (STIP1), histone deacetylase (HDAC), ATP-binding cassette transporter (ABC), and plasminogen activator inhibitor-1 (PAI-1). We also summarized the impact of intermediate target mutations on drug outcomes in Supplementary Table S1.

Glucocorticoid is a kind of CS classification, and NR3C1 is a gene that codes glucocorticoid receptor (GR). NR3C1 mutations can reduce the transcriptional activity of genes or even lose the activity of protein expression products, affecting GR’s formation and then impacting GR’s response to CS, resulting in CS resistance. Many studies have shown that NR3C1 is closely related to CS resistance in nephrotic syndrome (Ye et al., 2003; Liu et al., 2018; Parvin et al., 2021). These SNPs sites that mainly affect the sensitivity of GR include Tth111I, BclI, ER22/23EK, and N363S (Panek et al., 2015; Cheng et al., 2016).

CRHR1 is the key factor that mediates corticotropin-releasing hormone (CRH) to stimulate adrenocorticotropic hormone (ATCH) to secrete CS, which is indispensable in the secretion of human CS. If the CRHR1 gene is mutated, it can reduce or lose the gene’s function and inhibit the increased secretion of CS stimulated by inflammation by decreasing the secretion of ACTH. Therefore, the genetic variation of CRHR1 may be the most useful marker to identify the deficiency of endogenous CS and respond better to exogenous CS. Based on this theory, studies have shown that CRHR1 gene variants (including rs739645, rs1876831, rs1876829, and rs1876828) can enhance the efficacy of CS in asthma (Mougey et al., 2013).

Moreover, a variety of GLCCI1 mutated alleles are mainly associated with the efficacy of CS (Tantisira et al., 2011; Izuhara et al., 2014). Current studies have focused on asthma, where GLCCI1 gene variability is related to a noticeable decline in response to inhaled glucocorticoids in asthmatic patients (Tantisira et al., 2011). Current studies have focused on asthma, where GLCCI1 gene variability is related to a noticeable decline in response to inhaled glucocorticoids in asthmatic patients (Cheong et al., 2012). Whether this applies equally to IgA vasculitis is unknown.

STIP1 is a coordinated GR receptor chaperone protein that participates in CS binding and activating of intracellular GR complex and plays an anti-inflammatory role. STIP1 gene variation may regulate the CS response of asthma patients with impaired pulmonary function (Hawkins et al., 2009). Furthermore, HDAC is related to the activation mechanism of CS. The recruitment of HDAC2 is an essential step in the process of CS inhibiting inflammatory genes. In some diseases, the activity of HDAC2 is low, resulting in a weak answer to glucocorticoids (Barnes et al., 2004). Therefore, HDAC2 may become the target medium for predicting CS reactions in the future.

In addition, in idiopathic nephrotic syndrome pediatric, the 1236C > T polymorphism, one of the ABCB1 (belonging to ABC) gene mutations, may be associated with CS resistance, and the T allele and TT genotype may be closely associated with resistance and may require other therapeutic strategies (Safan et al., 2017). Here, we highlight the genetic polymorphisms associated with CS-induced osteonecrosis of the femoral head (ONFH), an essential gene for which several meta-analyses have shown its mutant phenotype (3435C > T) is a protective gene for CS-induced ONFH (Zhang et al., 2017; Chen et al., 2018; Lee et al., 2022). In other words, the T allele can significantly reduce the risk of CS-induced ONFH compared to the C allele. However, in a meta-analysis in 2015, ABCB1 rs1045642, the mutant phenotype (3435C > T), was an increased risk factor for the development of ONFH in women under the allele model. Still, this association was not found under the dominant model (Zhou et al., 2015). Another gene known to be associated with CS-induced ONFH is PAI-1. The well-studied mutant PAI-1 4G/5G (rs1799889) increases the risk of CS-induced ONFH development (Sobhan et al., 2018). A recent meta-analysis concluded that patients with 5G were at a lower risk than those with 4G during CS-induced ONFH development (Lee et al., 2022). Further studies are yet to be done in the future.

### 4.3 Immunosuppressants

#### 4.3.1 Cyclophosphamide

Cyclophosphamide (CYC) can inhibit the function of T cells and B cells and then reduce or prevent abnormal immune complexes sludging in glomeruli (Kanigicherla et al., 2018). CYC is one of the immunosuppressants with high-quality evidence and has been widely recognized by scholars at home and abroad for treating IgAV nephritis. As the only immunosuppressant recommended by KDIGO in 2012, CYC combined with CS can be used in children with crescentic IgAV nephritis complicated with nephrotic syndrome and/or renal function deterioration (KDIGO, 2012). Fortunately, with the continuous development of relevant research, the guidelines and consensus on using CYC recommendations are also more positive. The latest KDIGO guideline published in 2021 suggests that CYC should be used for IgAV nephritis with nephrotic syndrome or rapid deterioration of renal function (Rovin et al., 2021). IV CYC can be taken into account as second-line therapy for moderate IgAV nephritis and induction therapy (6–9 months) for severe IgAV nephritis (Ozen et al., 2019; Abu-Zaid et al., 2021), and second-line therapy for mild IgAV nephritis (Ozen et al., 2019). Considering the adverse reactions (ADR) of CYC, sequential maintenance therapy with other immunosuppressants after induction therapy is recommended. It is worth mentioning that the guidelines do not recommend oral CYC therapy because of insufficient evidence.

Although using CYC in IgAV nephritis has been more widely recognized and recommended, it varies significantly among individuals. The exposure of metabolites *in vivo* can be ten times different when ordinary people take the same amount of CYC (de Jonge et al., 2005). Moreover, the drug is prone to hemorrhagic cystitis, ovarian toxicity, leukopenia, male semen toxicity, and other ADR (Chong et al., 1983; Koetter, 2009; Rocha et al., 2009; Sharma et al., 2020). CYC is an inactive prodrug that needs to be metabolized into active 4-hydroxycyclophosphamide (4-OHCPA) through CYP2B6, 2C9, 2C19, and 3A4 *in vivo* (Roy et al., 1999), and then glutathione S-transferase (GST) will inactivate the latter. Besides, multidrug resistance-associated proteins (MRPs, such as MRP1 and MRP4) and ALDHs play an essential role in CYC transport and metabolism.

There are many types of CYP2B6 gene mutations, and studies have shown that many kinds of SNPs are related to their efficacy and safety. In terms of the coding region, CYP2B6*2 (c.64C > T) and CYP2B6*4 (c.785A > G) mutations increased enzyme activity, which significantly increased the risk of ADR (hemorrhagic cystitis and oral mucositis, respectively) for people with these allele mutations (Rocha et al., 2009). In contrast, CYP2B6*5 (c.1459C > T) mutation led to a decline in enzymatic activity and prominently increased the risk of reduced efficacy (Takada et al., 2004; Uppugunduri et al., 2014). Also, similar results have been obtained in breast cancer group patients treated with CYC, with CYP2B6*2, *4, *8, and *9 alleles related to poor clinical outcomes (Bray et al., 2010). Interestingly, the same CYP2B6 (c.516G > T) can increase or decrease enzyme activity in different researches (Xie et al., 2006; Joy et al., 2012), showing contradictory results. The conflicting results may be related to the diseases studied, the research methods, the mode of administration, the medication timing, and the medicine combination. Except for the coding region, a study in Japan found that CYP2B6 (g.−2320T > C, g.18492T > C, and g.−750T > C) in the non-coding region was also significantly associated with ADR (including leukopenia) of CYC (Nakajima et al., 2007).

The metabolism of CYC in CYP2C19 is less than that in CYP2B6, and it has a more negligible effect on the activation of CYC. CYP2C19*2 can induce a deficit of enzyme activity, which reduces the activation level of CYC and the risk of ADR (ovarian toxicity) (Singh et al., 2007; Tatarunas et al., 2014). Whereas, if the dose of CYC exceeds 1 g/m^2^, the mutation does not affect the metabolic level of CYC (Timm et al., 2005). It is speculated that the reason may be that the offset of CYP2B6 induced by CYC itself even exceeds the loss of enzyme activity caused by CYP2C19*2 (Martin et al., 2003). Oppositely, homozygous individuals carrying CYP2C19*1/*1 show an increased risk of ovarian toxicity (Ngamjanyaporn et al., 2011).

CYP2C9 and CYP3A4 play fewer roles in CYC activation. CYP2C9*2 (c.430C > T) and *3 (c.1075A > C) may increase the risk of leukopenia, but, interestingly, they may have a preferable effect on the therapeutic regimen (Schirmer et al., 2016). A better therapeutic response may be since the higher 4-hydroxylase activity of wild-type proteins than those encoded by CYP2C9*2 and *3, and the lower activity leads to slower activation of its intermediates by prodrug CYC (Timm et al., 2005). Notably, the formation of chloroacetaldehyde, a toxic metabolite of CYC, can be significantly reduced in combination with CYP3A4 inhibitors such as triptolide and ketoconazole, reducing the risk of nephrotoxicity and neurotoxicity (Zhang X. F. et al., 2014; Yang et al., 2018). This drug interaction seems to be beneficial to children.

Glutathione S-transferase (GST) is a phase II metabolic enzyme that can inactivate CYC and its intermediate metabolites, including GSTA1, GSTP1, GSTT1, and GSTM1. In a recent study on the mutation of GSTA1, it was found that compared with wild-type and heterozygous carriers carrying GSTA1 (−69C > T, rs3957356) T allele, patients with a homozygous mutation of GSTA1 (−69C > T, rs3957356) T allele were prone to unresponsiveness to CYC and more likely to be toxic (Attia et al., 2021). Increased risk of severe leukopenia was found in GSTA1*B carriers (Afsar et al., 2012). Compared with wild-type GSTM1 allele, only defective GSTM1 alleles are associated with more ADR (Hajdinak et al., 2020). Inversely, the GSTP1 I105V allele can reduce the enzyme activity, and the response rate of mutant individuals to CYC treatment is significantly higher. In addition, some studies have found that a mutation in ABCC4 (rs9561778) encoding MRP4 is significantly correlated with CYC-induced ADR (gastrointestinal toxicity and leukopenia/neutropenia) (Low et al., 2009).

#### 4.3.2 Mycophenolate mofetil and azathioprine

Mycophenolate mofetil (MMF) and azathioprine (AZA) are often regarded as immunosuppressants of the same recommended level in IgAV glomerulonephritis, and both are prodrugs. *In vivo*, a vital substance first converted from MMF is Mycophenolic acid (MPA). The MPA can downregulate the activity of inosine monophosphate dehydrogenase (IMPDH) in the critical pathway of purine synthesis, blocking the T and B lymphocytes proliferation and differentiation, thus effectively inhibiting the renal damage caused by abnormal immune reactions (Allison and Eugui, 2000). After entering our body, AZA is activated into cytotoxic thioguanine nucleotides. The latter can alleviate inflammation by restraining DNA replication and arresting CD28 co-stimulatory signals but will be inactivated by thiopurine methyltransferase (TPMT).

Compared with the 2012 KDIGO guidelines, the recommendations of immunosuppressants such as CYC, MMF, and AZA were more positive by subsequent evidence-based consensus or guidelines. In China, experts support CS combined with immunosuppressants in treating severe IgAV nephritis in children (Subspecialty Group of Renal Diseases and Chinese Medical Association, 2017). The recommended usage of MMF/AZA combined with CS was as follows: MMF 20–30 mg/kg/d, oral twice, gradually reduced after 3–6 months, and the entire course of treatment was 12–24 months, or the dose of AZA is 2 mg/kg/d for 8 months to 1 year. AZA and MMF can be taken into account as second-line therapy for mild IgAV nephritis and the first-line or second-line therapy for moderate patients. Considering the ADR of CYC, it is suggested that MMF or AZA should be the maintenance therapy after CYC induction treatment for the severe IgAV nephritis group (Ozen et al., 2019; Abu-Zaid et al., 2021). Besides CYC, KDIGO was skeptical about immunosuppressants, so no other immunosuppressants were recommended. The SHARE initiative and the consensus of Egyptian experts agreed that effective immunosuppressants could prevent/minimize the risk of renal kidney disease or renal failure, especially in rapidly progressive acute vasculitis (Ozen et al., 2019; Abu-Zaid et al., 2021), so the recommended intensity of immunosuppressants was also stronger. However, for MMF or AZA, which is more suitable for priority recommendation, there is a lack of corresponding research, and high-quality evidence is needed to distinguish the advantages and disadvantages.

MPA has biological activity as an intermediate from MMF and can inhibit IMPDH. It is catalyzed and transformed by uridine diphosphate glucuronosyltransferase (UGT). Most MPA is transformed by UGT1A9 into 7-O-MPA-glucuronide (7-O-MPAG) without activity, and a small part of MPA is transformed into Acyl-MPA-glucuronide (AcMPAG) by UGT2B7 (Picard et al., 2005). Remarkably, MPAG has the characteristic of the enterohepatic circulation process. Part of MPAG can be secreted into the bile and then converted into MPA in the intestine, which enters the body again after entering the blood through the portal vein. The transporters involved include organic anion transporting polypeptide (OATP) and multidrug-resistant protein-2 (MRP2) (Barraclough et al., 2010). In conclusion, IMPDH involved in the pharmacodynamics of MMF, UGT involved in metabolism, and OATP and MRP2 engaged in the transport process are related to the individual differences of MMF in patients.

It is reported that IMPDH can be divided into IMPDH type I and type II (Natsumeda et al., 1990). A study on renal transplant patients demonstrated that a mutation of IMPDH type II (3757T > C, rs11706052) was related to improving IMPDH bioactivity, which explained 8.0% of the patient-to-patient variance of IMPDH bioactivity (Sombogaard et al., 2009). The increase in IMPDH bioactivity is detrimental to MPA immunosuppression. Furthermore, studies have shown that IMPDH type I (rs2278293 and rs2278294) is related to a decrease in acute rejection 1 year after renal transplantation (Keown et al., 1996). Paradoxically, in an extensive research on 1,040 renal transplant patients, the results suggested that the above three IMPDH variants had no significant effect on acute rejection, MMF tolerance dose, 1-year allograft function, and 5-year allograft survival (Shah et al., 2012). The researchers believe that the main reason for the difference in the results may be longer follow-up (5 years) and a larger population cohort (1,040 patients) than in previous studies. Besides, they also found that patients carrying IMPDH type I (rs2278294) mutant A allele had a remarkable difference in the 2-year graft survival rate compared with other genotypes but was not proved at 5 years. It is the above research that demonstrated the essentiality of long-term follow-up. Of course, the differences in medical level and ethnicity between studies also contribute to differences in the results. Therefore, the current research results do not support the prior detection of IMPDH allele polymorphism in renal transplant recipients, let alone children with IgAV nephritis.

Among the mutants of UGT, UGT1A9 is the most studied. UGT1A9 is one of the most studied UGT variants. Firstly, the decrease in MPA exposure was found in the renal transplant population carrying the UGT1A9 (−2152C > T, rs17868320) or UGT1A9 (−275T > A, rs6714486) mutation genes located in the gene promoter region (Kuypers et al., 2005; Mazidi et al., 2013), which may be due to the blocking of the intestinal and liver circulation process of MPAG. Secondly, several studies have shown that in the same region of the gene coding UGT1A9, the other two SNPs (−440C > T and −331T > C) significantly impact the *in vivo* metabolic process of MPA in patients with renal transplantation (Baldelli et al., 2007; Levesque et al., 2008). However, the subjects are also renal transplant recipients. In a new Chinese renal transplantation study, researchers have not found that various SNPs of UGT1A9 are related to the pharmacokinetics of MPA or MPAG (Yang et al., 2021). This difference remains to be discussed. The sample size is too small, there are differences in the combination of drugs, and even the sampling methods may affect the study results. The polymorphisms of UGT1A1, UGT1A7, and UGT1A8, and the polymorphisms of MMF transporters including OATP, MRP2, ABCC2, and SLCO, all contribute to the individualized differences of MMF. Still, the evidence quality of clinical studies is not high enough to influence clinical decision-making.

AZA is prone to myelosuppression, mainly caused by excessive induction of thioguanine nucleotides, an intermediate metabolite. The prevailing view is that the TPMT alleles are critical in this step because mutations in the TPMT gene result in reduced or absent enzyme activity, causing a considerable accumulation of approximately 90% of thioguanine nucleotides that cannot be converted. Compared with adults, the TPMT activity of children is almost the same as that of adults (Schaeffeler et al., 2004). Although 40 variants of the TPMT allele (Appell et al., 2013) have been identified, more than 90% of the decrease in enzyme activity is mainly associated with TPMT*2 (c.238G > C), *3A (c.460G > A, c.719A > G), *3B (c.460G > A) and *3C (c.719A > G) polymorphisms. Patients with homozygous TPMT mutations had low TPMT activity. In contrast, patients with heterozygous TPMT mutations had moderate TPMT activity, and those without mutations had high TPMT activity. TPMT has good genotypic and phenotypic consistency (inconsistencies may be due to blood transfusion since TPMT is measured in red blood cells) (Yates et al., 1997). Myelosuppression is more prone in patients with low and moderate TPMT activity than in those with high TPMT activity. Nevertheless, the latter population needs more doses to resist rapid intermediate metabolite inactivation (Yates et al., 1997). Studies have shown that TPMT genotyping and dose adjustment before using AZA can reduce the incidence of ADR in intermediate metabolites to a level similar to normal metabolism in the Spanish population (Casajus et al., 2022).

Furthermore, mutations in the nucleoside diphosphate-linked moiety X-type motif 15 (NUDT15) gene have been reported as a more predictive predictor of AZA-induced leukocytopenia in Asian patients than TPMT. The low frequency of TPMT mutations in Asian populations may make NUDT15 more dominant in the use of AZA. A meta-analysis found that NUDT15*2 and *3 were critical genetic markers of early myelotoxicity elicited by thiopurine drugs (including AZA) in Asians (Khaeso et al., 2021). The NUDT15*3 mutant is a predictive factor for AZA-elicited myelosuppression in Chinese, so it has been suggested that patients carrying homozygous NUDT15*3 should avoid using AZA (Chen et al., 2021; Miao et al., 2021). Compared with TPMT, NUDT15 variant genotyping has higher accuracy in predicting AZA-induced leukopenia in the Indian population and can be used to optimize azathioprine dose (Banerjee et al., 2020).

In addition to the gene polymorphisms of TPMT and NUDT15, the allelic variation of inosine triphosphate pyrophosphatase (ITPA) during AZA metabolism also affects ADR. Most mutations in ITPA genes lead to a decrease in the bioactivity of ITPA, and the primary mutations are ITPA (c.94C > A, IVS2 + 21A > C, c.138G > A, IVS3+101G > A), in which c.94C > A pure and mutation almost inactivate the enzyme (Marsh et al., 2004; Simone et al., 2013). Low ITPA activity increases the risk of granulocytopenia, liver damage, or influenza-like symptoms, rash, fever, nausea, and vomiting (Simone et al., 2013; Wang et al., 2014). Nevertheless, a trial of about 550 adults with inflammatory bowel disease (IBD) showed that the ITPA allele has nothing to do with treatment-related ADR (Steponaitiene et al., 2016). Whether this is related to the sample size needs more research to prove the hypothesis.

#### 4.3.3 Cyclosporin A

Cyclosporin A (CyA) has rich application experience in various immune diseases and organ transplantation as a calcineurin inhibitor, but few studies have been conducted in children with IgAV nephritis. Koskela et al. (2019) studied 42 children with IgAV nephritis who used the MP and 20 children who used CyA and found that the long-term efficacy of the two groups was better. In the study, 5 years later, 38% of patients receiving MP treatment and 10% of patients receiving CyA treatment needed additional immunosuppressive therapy because of their condition. Therefore, Koskela et al. (2019) believed that these children with poor responses to MP maybe require long-term CyA therapy to inhibit the progression of IgAV nephritis. Jauhola et al. (2011) also followed up 24 children with severe IgAV for an average of 6 years and found that CyA treatment achieved the goal of complete remission of proteinuria faster than the MP group, while 6 of 13 MP patients needed additional immunosuppressive therapy, one of them had ESRD and received renal transplantation. This study suggests that CyA is more effective and safer than MP in treating severe IgAV. Studies have also shown that CyA may be effective in patients with CS resistance (Ohara et al., 2013). However, CyA treatment is easy to develop into CyA-dependent nephritis (Ronkainen et al., 2003; Park et al., 2011). Moreover, most researches have few cases and insufficient evidence, so the guidelines pointed out that CyA cannot be routinely recommended for moderate IgAV nephritis patients. However, CyA should be considered in patients with CS resistance or other immunosuppressants that are ineffective and relapsed.

The ADR of CyA includes hepatorenal toxicity, hypertension, neurotoxicity, gastrointestinal symptoms, and hairiness (Phan et al., 2021), especially hepatorenal toxicity showing a significant dose-effect relationship. CyA is biotransformed mainly by CYP3A4 and CYP3A5 into primary metabolites, including AM1, AM9, and AM4N, and then into secondary and tertiary metabolites AM1c, AM19, and AM1c9 (Lensmeyer et al., 1988; Christians et al., 1991a; Christians et al., 1991b). CyA principally relies on CYP3A4 metabolism *in vivo*, while a similar drug, Tacrolimus (TAC), is mainly metabolized by CYP3A5. Compared with CYP3A5, which mainly plays a role in the kidney, CYP3A4 metabolizes most CyA in the liver, and its variant CYP3A4*22 (c.522-191C > T, rs35599367) was related to the decline of enzyme bioactivity expressed in the liver, which is also related to individual clearance declining and renal toxicity (Elens et al., 2011; Wang and Sadee, 2016), such as worse creatinine clearance rate. As for CYP3A5, it is generally believed that carrying wild-type CYP3A5*1 allele (including homozygous and heterozygous) is considered CYP3A5 expression type, while CYP3A5*3 and *6 are considered to be CYP3A5 low expression or non-expression type due to incorrect mRNA splicing (Kuehl et al., 2001; Lin et al., 2002). Zheng et al. (2013) observed that individuals with CYP3A5 expression type (including homozygous and heterozygous) showed an enhanced formation of secondary metabolites AM19 and AM1c9, which may augment the risk of nephrotoxicity (Dai et al., 2004; Zheng et al., 2013).

Except for CYP3A4 and 3A5, P-gp and P450 oxidoreductase genes (POR) also have affected the pharmacokinetics (PK) of CyA. As one of the sensitive substrates of P-gp, the bioavailability of CyA is low. P-gp, a member of the ABC membrane transporter family (including ABCC2 and ABCG2), is a product of human multidrug resistance gene 1 (MDR1). It has been discovered that adults carrying the POR*28 allele require more CsA dose (Cvetkovic et al., 2017), while ABCC2 (c.-24C > T, rs717620) and ABCG2 (c.421C > A, rs2231142) may increase the exposure of CyA *in vivo*, which in turn increases the risk of hepatorenal toxicity (Wang et al., 2021).

#### 4.3.4 Other immunosuppressants

##### 4.3.4.1 Tacrolimus

TAC is a widely used immunosuppressive drug whose mechanism of action is to bind to the cytosolic receptor FK-binding protein 12 in T lymphocytes to form a composite that combines with calcium-regulated neurophosphatase to prevent dephosphorylation and nucleus translocation of nuclear factors in inflamed T cells, eventually inhibiting the expression of interleukin 2 and activation of T lymphocytes (Bowman and Brennan, 2008). The demethylation and hydroxylation occur *via* CYP3A isoforms (CYP3A4 and CYP3A5) in the hepar and intestine, while TAC is the substrate of the multidrug efflux transporter P-glycoprotein which is epitomized by the ABCB1 gene and previously known as MDR1; and it is represented on a multitude of epithelial cells, endotheliocyte, and lymphocytes (Birdwell et al., 2015). The restricted therapeutic index and pharmacokinetic differences between patients with TAC are partly due to the CYP3A5 gene variation, and CYP3A5 is the primary enzyme of TAC metabolism (Dai et al., 2006). Although TAC is currently used empirically in the treatment of childhood IgAV nephritis (Ichiyama et al., 2017; Zhang et al., 2018) and is believed to be a prospective non-steroidal medicine for treating severe IgAV nephritis, proof-based clinical information available are still modest. As a calcium-regulated neurophosphatase inhibitor (CNI), the critical mechanistic effects of TAC in childhood IgAV nephritis include inhibition of calcium-regulated neurophosphatase redistribution in the cleft septum, which may potentially reduce proteinuria (Namgoong, 2020). TAC has been proposed to potentially alleviate proteinuria through stabilization of the podocyte cytoskeleton primarily by inhibiting the expression of calcium-regulated neurophosphatase (Zhang et al., 2012). A recent retrospective clinical study concluded that TAC for the treatment of IgAV nephropathy in children was shown to reduce proteinuria, hematuria and improve renal function with relatively mild side effects, with major adverse events likely to include respiratory and urinary tract infections (Wu et al., 2022). The evidence for the safety of TAC in the treatment of IgAV nephritis in children is insufficient, however, if proteinuria persists after a course of CS, tacrolimus may also be considered for the immunosuppressive treatment of children with IgAV nephritis, with attention to the detection of relevant indicators and clinical symptoms in the child at the time of use.

The CYP3A5 expression phenotype exhibits negative or mildly decreased levels of CYP3A5 mRNA and expressed analogous vasoactive protein CYP3A5; in contrast, the CYP3A5 non-expression phenotype expresses low expression of the vasoactive CYP3A5 protein due to a CYP3A5*3 allelic variant encoding an enzyme with reduced activity (Hustert et al., 2001; Kuehl et al., 2001). This variant is most common in whites, with 80%–85% of whites being CYP3A5 non-expression (Van Schaik et al., 2002). Zhang et al. (2018) performed a study on the use of TAC beyond instructions in children with IgAV nephritis. They found that children with the CYP3A5*1 allelic variant had significantly lower dose-adjusted trough concentrations than children with the CYP3A5*3/*3 allelic variant. However, there was no difference in short-term efficacy between the two groups, probably due to the small sample size. Massive retrospective studies of current TAC use in renal transplant patients have shown that CYP3A5 expression renal transplant recipients require approximately twice the dose of TAC than non-expression patients (Birdwell et al., 2012). A controlled trial indicated that patients carrying the CYP3A5*1/*1 or CYP3A5*1/*3 genes (CYP3A5 expression) required an equal dose of TAC compared to patients carrying the CYP3A5*3/*3 genes (CYP3A5 non-expression) patients, with comparable blood intensities requiring 1.5 to 2 times the dose (Haufroid et al., 2004). And in the immediate first year after kidney transplantation, patients carrying the CYP3A5*3/*3 gene had 1.8–2.5 fold higher dose-adjusted trough concentrations than patients expressing CYP3A5 (Rojas et al., 2015). This proved that patients with CYP3A5 expression phenotypes metabolize TAC more strongly than patients with CYP3A5 non-expression phenotypes and that the required dose to maintain the target blood concentration should be higher. Based on the impact of CYP3A5 genotyping on the pharmacokinetic parameters of TAC, the guideline (Birdwell et al., 2015) gives recommendations for the dose of TAC to be used, with CYP3A5 expression patients will demand an upper recommended initial dose, while patients who do not express CYP3A5 will demand the standard recommended initial dose, and for faster achievement of therapeutic drug concentrations, it is recommended that taking TAC CYP3A5*3 allele testing is recommended for patients taking TAC prior to initiation of TAC. Also, considering risks of arterial constriction, hypertension, and renal toxicity that may develop with supra therapeutic concentrations of TAC, dose adjustment based on observed serum concentrations should be made for intermediate (CYP3A5*1/*3) or rapid (CYP3A5*1/*1) metabolizers using TDM.

There is still a lack of evidence that CYP3A5 genotyping guides the efficacy of TAC dosing in pediatric IgAV nephritis patients, and CYP3A5 genotyping does not replace therapeutic TDM due to the existence of many other factors that influence the dosing requirements of TAC, such as drug interactions with glucocorticoids and immunosuppressants commonly used in the treatment of IgAV nephritis and genetic variants that affect TAC pharmacodynamics. The two methods should be used in combined for individualized treatment of pediatric IgAV nephritis patients. Future clinical trials on the affection of CYP3A5 genotyping in guiding the effectiveness of using TAC in pediatric IgAV nephritis patients are needed, which will be of great significance for the precise dosing of TAC in pediatric IgAV nephritis patients.

##### 4.3.4.2 Mizoribine and leflunomide

Mizoribine (MZB) is an antimetabolic drug that exerts immunosuppressive effects by inhibiting lymphocyte proliferation (Yokota, 2002). The reported immunosuppressive effect of MZB is due to the suppression of DNA in the S phase of the cell cycle. There are clinical cases of using MZB instead of CYC in combination with pulsed MP as part of the treatment of severe IgAV nephritis in children, and this therapy has shown improvement in histological severity as well proteinuria in children with IgAV nephritis (Kawasaki et al., 2011). A clinical trial using MZB as a complementary therapy to prednisolone monotherapy in patients with IgAV nephritis showed that MZB may have a prophylactic effect in patients with IgAV nephritis at risk of relapse (Kawakami et al., 2010). MZB is a reliable and well-tolerated drug, and no significant adverse effects, including bone marrow suppression, have been reported in the treatment of children with IgAV nephritis. This suggests that MZB is likely to be a potential drug for managing IgAV nephritis in children.

In clinical experience, many patients and their families are reluctant to undergo treatment with CTX primarily because of its gonadal toxicity. The high cost of MMF may have an impact on treatment choice. Leflunomide (LEF) is a comparatively new oral immunosuppressant that disrupts T and B cell function by inhibiting dihydronucleic acid dehydrogenase (the rate-limiting enzyme for ab initio synthesis of pyrimidine nucleotides), specifically inhibits T-dependent autoantibody formation; and a few tyrosine kinase signaling molecules involved in immune function are also inhibited by LEF (Siemasko et al., 1996; Lou et al., 2006). The current clinical trial results showed that LEF combined with steroids improved the renal prognosis of patients with IgAV nephritis with nephrotic proteinuria more significantly than steroids alone, with no apparent adverse effects (Zhang Y. et al., 2014; Hou et al., 2021). LEF, which has been reported to be rapidly biotransformed to its active metabolite, is usually undetectable in plasma and therefore has only a tiny *in vivo* exposure (and no pharmacological activity). *In vitro* studies suggest that activation of LEF may be associated with CYP4501A2, CYP2C19, and CYP3A4 (Rozman, 2002; Kalgutkar et al., 2003), and only limited pharmacogenetic data are available for LEF or its active metabolite. When LEF is used for the treatment of rheumatoid arthritis (RA), individuals carrying the CYP1A2*1F CC gene are more likely to have a 9.7-fold higher risk of overall toxicity than those carrying the CYP1A2*1F A allele (Bohanec Grabar et al., 2008). Furthermore, ABCG2, as an efflux transporter, is involved in the disposal of various chemotherapeutic drugs, and it has been reported that LEF and its active metabolites are substrates of ABCG2 *in vitro* (Kis et al., 2009). So ABCG2 gene polymorphism may be a major determinant of interindividual differences in the disposition of the active metabolite of LEF-teflunomide. A study investigated the effect of the ABCG2 (c.421C > A and c.34G > A) gene on teflunomide PK in 24 healthy volunteers and concluded that ABCG2 c.421C > A polymorphism seems to be the main determinant of inter-individual pharmacokinetic differences in teflunomide (Kim et al., 2011). Overall, LEF can be considered an alternative drug with good efficacy and safety profile in pediatric patients with IgAV nephritis who are intolerant to commonly used immunosuppressive agents.

##### 4.3.4.3 Monoclonal antibodies

Rituximab (RTX), a monoclonal antibody against the B-cell CD20 antigen, was initially conceived and endorsed in 1997 for the therapy of non-Hodgkin’s B-cell lymphoma and, as such, has been used in many immune-mediated diseases, for example, has become the standard therapy for inducing remission and maintaining resolution in patients with anti-neutrophil cytoplasmic antibody (ANCA)-associated vasculitis (Modena et al., 2013; Clark, 2020). Studies evaluated the major lymphocyte subsets in the peripheral blood of children with IgAV and showed an increased percentage of B lymphocytes as well as increased serum IgA in children with IgAV (Wiercinski et al., 2001), and RTX, by depleting B cells, may reduce the formation of IgA-containing immune complexes and limit the activity of IgA disease (Fenoglio et al., 2017). Although RTX should not deplete terminally differentiated, immunoglobulin-secreting plasma cells, the benefit of depleting B cells by RTX in other B cell-mediated diseases guarantees its use in refractory IgAV (Donnithorne et al., 2009). RTX has therefore been suggested as an alternative therapy for refractory or recurrent IgAV (Abu-Zaid et al., 2021) and is a potential therapeutic tool for IgAV nephritis that is attractive in refractory IgAV nephritis.

In case reports with adult patients with IgAV nephritis (Pindi Sala et al., 2014; Bellan et al., 2016; Fenoglio et al., 2017), RTX was primarily used as a salvage treatment for the onset of IgAV nephritis, where patients received RTX for refractory/recurrent disease or because conventional CS/immunosuppressive therapy was contraindicated, and complete renal remission was achieved after treatment, during which all patients were in sustained remission. No serious adverse events associated with RTX were usually observed, and only a minimal number of patients experienced gastrointestinal norovirus infection in patients 3 months after RTX. However, this infection may have been associated not only with RTX treatment but also with other immunosuppressive therapies previously received. Meanwhile, a report by Katherine et al. (Donnithorne et al., 2009) demonstrated the effectiveness of RTX in children with severe IgAV nephritis who had failed to respond to conventional interventions. A clinical study on IgA nephropathy (IgAN) patients conducted by Richard et al. (Lafayette et al., 2017) yielded different results, with RTX treatment failing to significantly reduce proteinuria or improve renal function in IgAN patients during the period studied. When RTX was used for renal disease, the older the patient was used, the better the outcome, such as the lower risk of relapse, longer B-cell recovery, and lower risk of hypogammaglobulinemia; however, these associations have not been universally confirmed (Sinha et al., 2021). Overall, RTX is a potential therapeutic agent for severe childhood IgAV nephritis where conventional interventions have failed. However, pediatric data regarding the use of RTX for IgAV nephritis in children are still sparse. The lack of randomized controlled trials is insufficient to conclude that RTX is equally secure and effective in pediatric patients, and more clinical data are needed.

### 4.4 Others

#### 4.4.1 Complement inhibitors

The complement system, a critical player in the body’s response to defense, exerts a powerful effect upon many physiological systems. However, unintentional activation of the complement system may turn the scales in favor of self-aggression, leading to an over-reaction to self-aggression and auto-inflammatory illnesses (Ort et al., 2020), where the kidney is particularly vulnerable, and abnormal complement activation is associated with many renal diseases. Patients with IgAV have an increased number of IgA immune complexes that are deposited on the glomerular thylakoid because of the lack of C1q binding sites IgA fails to activate the classical pathway of complement but activates the complement system *via* the alternative or lectin pathway (Chen and Mao, 2015; Heineke et al., 2017), ultimately leading to podocyte and glomerular injury, fibrosis and ESRD (Ort et al., 2020).

The availability of eculizumab (eculizumab) in 2007 demonstrated that complement inhibitors could treat rare (auto) inflammatory diseases (Ort et al., 2020). Many next-generation complement inhibitors, including humanized monoclonal antibodies, small proteins that bind to specific complement components, and recombinant proteins, are currently being evaluated in clinical trials (Zipfel et al., 2019). Mannan-binding lectin-associated serine protease 2 (MASP-2), an effector enzyme activated by the complement lectin pathway, is a potential drug target for IgAV nephritis (Lafayette et al., 2020). Narsoplimab, a fully human monoclonal antibody, is designed to treat diseases mediated by the complement agglutinin pathway by inhibiting of MASP-2. The classical complement activation pathway, which is not interfered with by inhibition of MASP-2, plays a vital role in the immune response to acquired infections (Lafayette et al., 2020); therefore, the use of narsoplimab has not increased the risk of infection or autoimmune disease. Currently, guidelines suggest that patients need meningococcal vaccination and appropriate prophylaxis when complement deficient or treated with complementing inhibitors (Rovin et al., 2021).

The current results of many basic studies support the idea that complement activation can play a critical role in the development and progression of IgAN disease; for example, narsoplimab appears to significantly reduce proteinuria and increase the stability of EGFR in renal function in high-risk IgAN patients (Edström Halling et al., 2010). Since the pathophysiology of IgAV nephritis is similar or identical to that of IgAN (Chen and Mao, 2015) and is also accompanied by IgA deposition, complement factors, and massive neutrophil infiltration (Heineke et al., 2017), narsoplimab would be equally very promising as a complement inhibitor in patients with IgAV nephritis.

#### 4.4.2 Anticoagulants and antiplatelet drugs

Abnormalities in the coagulation and fibrinolytic systems can contribute to the development of IgAV nephritis (Prandota et al., 2001); and activation of intra-glomerular coagulation is an aggravating factor in IgAV nephritis, which may be related to fibrin deposition in the glomerulus contributing to the crescent formation (Shin et al., 2005). In experimental models, defibrinolysis reduced the number of crescent bodies and the severity of renal insufficiency (Tipping et al., 1986). Therefore, the use of appropriate fibrinolytic and anticoagulant drugs may have significance for preventing and treating IgAV nephritis in children. Although still controversial, evidence-based guidelines in China suggest that anticoagulants and/or antiplatelet aggregating agents can be used to treat IgAV nephritis (Subspecialty Group of Renal Diseases and Chinese Medical Association, 2017). Common anticoagulants and antiplatelet agents, including warfarin, heparin, pentoxifylline, and aspirin (Chartapisak et al., 2009), and the fibrinolytic drug urokinase.

Iijima et al. (1998) suggested that various combinations of glucocorticoids and anti-immune drugs with heparin/warfarin and pansentin are effective in histologically severe IgAV nephritis. However, a recent case report (Kalay-Yildizhan et al., 2020) reported the development of skin ulcerated plaques and proteinuria in IgAV patients treated with warfarin, as well as a rare complication with heparin, namely heparin-induced thrombocytopenia (HIT), which activates platelets and leads to thrombocytopenia and a prothrombotic state. Treatment with anticoagulants in patients with IgAV should be vigilant for these rare but serious consequences, and immediate discontinuation of the drug is essential to prevent renal failure and other organ involvement. Watanabe et al. (Kawasaki et al., 2004b) affirmed urokinase’s efficacy in treating severe IgAV nephritis and recommended fibrinolytic therapy to alleviate the intra-glomerular hypercoagulable state resulting from renal injury to protect renal function in patients with severe IgAV nephritis. A clinical trial (Kawasaki et al., 2003) showed that hypercoagulable in IgAV nephritis improved with urokinase shock treatment compared to pre-treatment, with lower urinary protein excretion and lower volume of cases of renal disfunction after treatment, and no ADR such as bleeding tendency were observed. Meanwhile, a study (Shin et al., 2005) found that children with IgAV nephritis who develop the disease at an age older than 9 years have relatively severe fibrinogen deposition, suggesting that age of onset affects the process and prognosis of fibrinogen deposition. Moreover, childhood IgAV nephritis is a heterogeneous disease with multiple histopathological manifestations. The increase of crescent bodies in children with IgAV nephritis is not always dependent on fibrinogen, so fibrinolytic therapy should be tailored to the child’s characteristics to achieve individualized treatment.

#### 4.4.3 Chinese medicine

Chinese medicine treatment has been considered an effective treatment for children with IgAV nephritis; its combination with immunosuppressants and CS produces additional positive effects, such as alleviating blood hypercoagulability in children, reducing proteinuria, and decreasing the recurrence rate of IgAV nephritis in children (Ding et al., 2019; Jin et al., 2021). Tripterygium wilfordii, a vine grown in southeastern China for thousands of years, is the most widely used Chinese medicine in children with IgAV nephritis. Tripterygium glycosides, a natural active ingredient extracted from tripterygium wilfordii, could inhibit delayed metabolic reactions, reduce the secretion of cytokine IL-1, and inhibit the function, division, and replication of T lymphocytes (Wang et al., 2019). Furthermore, the tripterygium glycosides antagonize the binding of inflammatory factors to receptors, inhibiting the release of inflammatory transmitters (Han et al., 2016). The ability of tripterygium glycosides to alleviate hematuria and proteinuria in IgA deposition nephropathy has been demonstrated in a previous study (Liu X. et al., 2019). Tripterygium glycosides also possess some anti-inflammatory effects, and their combination with TAC can reduce the dosage of TAC, thus reducing the risk of complications such as hepatotoxicity and neutropenia. However, the time required to treat IgAV nephritis in children is longer than that of TAC alone, which may be related to the slow onset of action of the herbal medicine (Zhang et al., 2021). ADR to tripterygium glycosides is widespread and can involve multiple systems, including hematologic, cardiovascular, and neurologic. Although several clinical reports demonstrate favorable effects of tripterygium glycosides in children with IgAV nephritis for whom conventional therapy is ineffective, they are still insufficient to verify the safety of tripterygium glycosides. Based on the safety considerations of pediatric use, the current drug instructions for tripterygium glycosides explicitly indicate that it is prohibited in children. Therefore, the Chinese expert group did not recommend tripterygium glycosides for the treatment of IgAV nephritis in children in the 2017 guidelines (Subspecialty Group of Renal Diseases and Chinese Medical Association, 2017). As a potential drug for the treatment of IgAV nephritis in children, it is expected that more relevant randomized controlled clinical studies and prolonged follow-up will emerge in the future to help the decision on the use of tripterygium glycosides.

## 5 Non-drug treatment of immunoglobulin A vasculitis nephritis in children

### 5.1 Plasmapheresis

Plasmapheresis (PP) has become a typical extracorporeal circulation blood purification therapy, and studies on the treatment of IgAV nephritis in children with PP or PP combined with drug therapy have proved beneficial.

Hattori et al. (1999) retrospectively evaluated 9 cases of rapidly progressing IgAV nephritis in children treated with PP as the only treatment. All the children had proteinuria and decreased GFR, and renal biopsy indicated that the crescent shape was over 50%. The PP regimen was three times weekly for 2 weeks and then once a week for 6 weeks. The study showed that all patients responded quickly to PP treatment, with clinical symptoms improving, proteinuria decreasing, GFR improving, purpuric rash, and abdominal pain alleviating. Kawasaki et al. (2004a) retrospectively studied six rapidly progressive IgAV nephritis cases in children treated with PP combined with multiple drugs. PP regimen for all patients consisted of five courses for 2 weeks, three times every other day in the first week and two times every other day in the second week. No increase in urinary protein and crescents, glomerulosclerosis, and renal failure was observed during multidrug therapy after PP treatment, which has been shown to be beneficial for rapidly progressive IgAV nephritis in children. Liu N. et al. (2019) studied the clinical therapeutic effect and reliability of dual filter plasma exchange (DFPP) combined with drugs to treat severe IgAV nephritis in children. All children had severe IgAV nephritis and received DFPP for three courses on the basis of the drug treatment regimen of MP and CYC dual pulse therapy. The decrease in urinary protein, immunoglobulin, serum creatinine, and urea nitrogen was notably higher than those in the control group. There was non-differentiation in the incidence of ADR. The study results confirmed that DFPP combined with drugs could improve clinical symptoms and reduce renal injury without increasing the incidence of side effects.

The mechanism of PP treatment for IgAV nephritis in children is still unclear. The results of test indicators and immunology show that PP can remove IgA-containing immune complexes or accumulations of pro-inflammatory mediators, and reduce fibrous protein or other blood coagulable factors. It is speculated that these mechanisms are related (Kawasaki et al., 2004a). Overall, early PP treatment can effectively improve the prognosis of children with IgAV nephritis, especially in children with rapid progression and severe IgAV nephritis, which can significantly improve urinary protein, GFR, and other indicators, prevent and reduce renal injury, reduce purpura and other clinical symptoms (Kawasaki, 2011).

### 5.2 Tonsillectomy

Several current studies have shown that tonsillectomy is not only related to the reduction of urinary protein excretion but also can effectively treat IgAV nephritis in children and prevent the recurrence of IgAV nephritis in children (Yang et al., 2016; Umeda et al., 2020). There was a case of IgAV nephritis in children with ISKDC VI-grade. After tonsillectomy, the proteinuria was significantly reduced, and the renal pathological results were improved in this patient. Tonsillectomy was performed on 5 cases of IgAV nephritis in adults and children with ISKDC grade II-VI (Ohara et al., 2011). The study showed that the pathological results of renal biopsy after tonsillectomy were improved with or without methylprednisolone pulse therapy, and the prognosis was good, confirming that tonsillectomy can successfully treat IgAV nephritis in children with severe ISKDC grade VI. A study (Umeda et al., 2020) retrospectively analyzed 71 cases of IgAV nephritis in Japanese children, 31 of whom underwent tonsillectomy after methylprednisolone pulse therapy and 40 of whom received methylprednisolone pulse therapy alone. The study showed that 2 years after methylprednisolone pulse therapy, the recurrence rate of IgAV nephritis in children undergoing tonsillectomy was significantly lower than that in children without tonsillectomy, which confirmed that tonsillectomy was conducive to preventing the recurrence of IgAV nephritis in children.

The mechanism of tonsillectomy in the treatment of children with IgAV nephritis is not yet clear. According to the remarkable influence of tonsillectomy on proteinuria in children’s IgAV nephritis, it can cut down the production of IgA and the aberrant immunoreaction and inflammation after glomerular deposit IgA, which is different from the IgA immune compound removed by PP, suggesting that tonsil may act the part of the pathogenesis of IgAV nephritis (Kawasaki et al., 2006). On the other hand, chronic bacterial colonization of the upper airway or chronic inflammation has been considered to be the triggering factor of IgA vasculitis, and intervention measures to remove major lymphatic organs and reduce the recurrence of chronic inflammation may be helpful in reducing the induction of IgAV (Kronbichler et al., 2015). Therefore, tonsillectomy can significantly reduce the occurrence of proteinuria and hematuria and improve the pathological results of renal biopsy substantially, and other aspects the benefits. Tonsillectomy is also a good choice for treating IgAV nephritis in children, especially severe pathological grade IgAV nephritis, and prevention of recurrence of IgAV nephritis.

## 6 Discussion

IgAV nephritis is mainly due to renal involvement caused by aberrant o-glycosylation of IgA1 deposition in the renal. The ultimate object of its therapy is to relieve or even eliminate the long-term harm of IgAV to the kidney. Drug therapy for IgAV nephritis includes ACEI/ARBs, CS, CYC, MMF, and AZA. The evidence quality of these drugs is high, and the guidelines recommend. Potential drug therapy includes CyA, TAC, MEB, LEF, RTX, complement inhibitors, anticoagulant antiplatelet drugs, and traditional Chinese medicine. Non-drug therapy is mainly PP and tonsillectomy, which can be used as an alternative treatment or potential treatment for drug-resistant cases, recurrent cases, and refractory cases. This paper also emphatically discusses the therapeutic significance of CS and immunosuppressants on children with IgAV nephritis at the level of pharmacogenomics and analyzes the individualized application of CS and immunosuppressants on children with different genotypes to provide a more comprehensive reference for individualized treatment of children with IgAV nephritis. Pre-identification of genes that affect drug efficacy and toxicity can predict the response of individual patients to drug treatment in advance, thus helping to determine a more effective initial dose and maintenance dose and even choose a more appropriate treatment for IgAV nephritis. It can also provide genetic evidence for post hoc analysis of drug resistance and ADR. The implementation of pharmacogenomics detection can help many Chinese children achieve better treatment outcomes (Qin et al., 2020). Therefore, this article focuses on linking pharmacogenomics and children with IgAV nephritis, which will draw more attention and thinking about the precise dosing of immunosuppressants in children with IgAV nephritis. We will also pay more attention to the pharmacogenomics related to the use of CS and immunosuppressants and encourage you to actively explore the pharmacogenomics of CS and immunosuppressants in children with IgAV nephritis.

CS-related gene polymorphism is associated chiefly with CS resistance, especially NR3C1 gene polymorphism, but there is a lack of related research in patients with IgAV nephritis, especially in children. Therefore, NR3C1 and other related gene polymorphisms on CS responsiveness in other diseases apply to IgAV glomerulonephritis, which still needs further research to confirm its universality.

Most CYC-related genetic variations are associated with ADR, such as the higher risk of hemorrhagic cystitis and leukopenia associated with CYP2B6 and GST gene polymorphism (Rocha et al., 2009; Afsar et al., 2012). Unfortunately, these results of most related pharmacogenomics studies about CYC have poor repeatability and cannot be applied to clinical practice.

The most significant advantage of MMF is that it is safer than other immunosuppressants, and its gene polymorphism is more associated with curative effects than toxicity and is often studied in patients with renal transplantation. The research is still immature. For example, the research results in IMPDH and UGT1A9 have some contradictions and differences.

The Clinical Pharmacogenetics Implementation Consortium (CPIC) suggested that TPMT deficiency was detected by identifying TPMT allele variants or TPMT phenotypes associated with the inactivation of AZA active metabolites and gave strong dose recommendations for the use of AZA in IBD patients (Relling et al., 2011; Woillard et al., 2017). These recommendations also apply to children (Relling et al., 2013). In the updated 2018 CPIC guidelines, AZA dosage guidance based on TPMT activity is no longer limited to a few diseases; CPIC has extrapolated it to all diseases. In some non-malignant cases, those with moderate TPMT activity and low TPMT activity may avoid AZA in favor of other alternative drugs (Relling et al., 2019). For high TPMT activity, use the standard starting dose recommended according to the guidelines for the respective disease. For patients with moderate TPMT activity and low TPMT activity who must use AZA, a dose reduction of 30%–80% for moderate TPMT activity and a 10-fold decrease in daily dose for low TPMT activity is recommended (Relling et al., 2019). In addition to TPMT, the NUDT15 gene polymorphism is more instructive in Asians. Similar to AZA, regular starting doses are used for those with normal NUDT15 metabolism (i.e., NUDT15*1 pure-hybrid individuals) and those with intermediate NUDT15 metabolism (i.e., NUDT15*1-containing heterozygotes), starting doses should be reduced by 30%–80% (Relling et al., 2019). In contrast, those with poor NUDT15 metabolism (not carrying the NUDT15*1 allele) should substantially lessen the regular initial dose or avoid AZA (Relling et al., 2019). The initial dose should be determined according to disease-specific and race-specific guidelines. Furthermore, an initial screening for TPMT and NUDT15 can predict the drug response in advance, give an appropriate initial dose, and be used for post-mortem detection of severe myelotoxicity, liver failure, and drug resistance to find the genetic causes of ADR and drug resistance (Woillard et al., 2017). If the child has a homozygous mutation of TPMT or NUDT15, MMF can be selected in advance instead of AZA for treatment. If the child has a heterozygous mutation, a low dose of AZA is more recommended.

Several studies have shown that CYP3A4, CYP3A5, POR, ABC, and other gene polymorphisms are related to the individual differences of CyA to a certain extent, and they all can become factors that predict CyA response in advance. Unfortunately, the uncertainty of efficacy and toxicity of CyA in the IgAV population is an indispensable reason limiting the expansion of such research in the past and future.

Like AZA, the research on the relationship between gene polymorphism of TAC metabolizing enzyme CYP3A5 and drug dose is relatively mature in the renal transplantation population. Therefore, to use TAC more safely, it is recommended that prior CYP3A5 typing be performed before some solid organ transplants such as heart, kidney, and lung transplants (Woillard et al., 2017). CPIC also strongly proposed that patients with CYP3A5 non-expression start treatment of the standard commended dosage (0.15 mg/kg/d), while patients with CYP3A5 expression increase the initiation dosage to 1.5–2 times the commended initiation dosage to guide dosage adjustment by therapeutic drug monitoring (Birdwell et al., 2015). However, these are not recommended for children with IgAV nephritis.

For the objects of gene detection, there is no guiding principle for gene detection of children with IgAV nephritis, and there is no relevant research. However, according to the AZA genotyping guidelines (Woillard et al., 2017), genetic post hoc probing and validation should be performed when patients present with drug resistance, serious adverse effects (e.g., myelosuppression and nephrotoxicity), and abnormal blood levels values. Of course, ex-ante genotyping should be considered when patients are planning to use high-quality evidence like AZA and TAC and have genotyped dosing recommendations in other diseases to seek safe and effective initial and maintenance doses.

There are still some limitations here. The most important of these is the lack of drug-related genetic polymorphism studies in children with IgAV nephritis, which are currently limited to studies in other diseases, such as AZA in IBD and TAC in the renal transplant population, and the paucity of authoritative guidelines for patient genotype-based drug use and clinical evidence for physicians and pharmacists. Indeed, genetic testing increases the cost of treatment for children. It is unclear whether the increase in treatment cost is proportional to the improvement in therapeutic efficacy and safety. Despite the relative maturity of genetic testing technology, many medical institutions have not been able to implement this technology, which is related to the lack of pharmacy technicians, small patient populations, and physician attitudes toward genetic testing.

In conclusion, it may not be the best time for pharmacogenomics to guide the dosing of children with IgAV nephritis, but with the vision of precision dosing, our attention to pharmacogenomics should not be diminished, especially for drugs like AZA, which can already guide dosing based on patient genotype. Therefore, the future treatment of IgAV nephritis in children urgently needs high-quality pharmacogenomics-related clinical trials to provide more evidence-based medical and pharmacological evidence for clinical diagnosis and treatment.
