# Supplementary material for: Individualized medication based on pharmacogenomics and treatment progress in children with IgAV nephritis
Source: Front Pharmacol. 2022 Jul 22;13:956397. doi: 10.3389/fphar.2022.956397 (PMC9355498; doi:10.3389/fphar.2022.956397)
Supplement: Supplementary file 1 [file Table1.DOCX]

Supplementary Material

**Table S1: Key targets and mutations affecting the efficacy and safety of IgAV therapy.**

| **Drug** | **Target** | **Genetic variant** | **Significant impact (all Results for other diseases)** | **Clinical value** |
| --- | --- | --- | --- | --- |
| **CS** | NR3C1 | rs10052957  rs258751 | - related to CS resistance |  |
|  | CRHR1 | rs739645  rs1876831  rs1876828 | - cause insufficient endogenous CS   production |  |
|  | GLCC1 | rs37972  rs37973 | - reduce CS efficacy |  |
|  | STIP1 | rs4980524  rs6591838  rs2236647 | - increase responsiveness to CS   treatment |  |
|  | HDAC | rs1741981 | - reduces CS efficacy |  |
|  | ABCB1 | rs1128503 | - 1236C>T is related to CS resistance |  |
|  |  | rs1045642 | - 3435C>T reduces the risk of ONFH |  |
|  | PAI-1 | rs1799889 | - increases the risk of ONFH |  |
| **CYC** | CYP2B6 | rs8192709 (CYP2B6*2, c.64C>T)  rs2279343 (CYP2B6*4, c.785A>G) | - increase enzyme activity - increase the risk of ADR |  |
|  |  | rs3211371 (CYP2B6*5, c.1459C>T) | - reduces enzyme activity - affects the curative effect of CYC |  |
|  |  | rs2279345 (CYP2B6, g.18492T>C)  rs4802101 (CYP2B6, g.-750T>C) | - significantly related to ADR |  |
|  | CYP2C19 | rs12769205 (CYP2C19*2, 12662A>G)  rs4244285 (CYP2C19*2, 19154G>A) | - lack of enzyme activity - reduce the risk of ovarian toxicity |  |
|  |  | CYP2C19*1/*1 (wild-type) | - increases the risk of ovarian toxicity |  |
|  | CYP2C9 | rs1799853 (CYP2C9*2, c.430C>T) rs1057910 (CYP2C9*3, c.1075A>C) | - increase the risk of leukopenia - have a better response to treatment |  |
|  | GST | rs3957356 (GSTA1, -69C>T) | - related to ADR |  |
|  |  | rs1695 (GSTP1 I105V) | - lower enzyme activity |  |
| **MMF** | IMPDH | IMPDH type I: rs2278293, rs2278294 | - controversy: reduce acute rejection |  |
|  |  | IMPDH type II: rs11706052 | - increases activity of IMPDH |  |
|  | UGT | rs17868320 (UGT1A9, -2152C>T) rs6714486 (UGT1A9, -275T>A) | - reduce MPA exposure |  |
| **AZA** | TPMT | rs1800462 (TPMT*2, c.238G>C)  rs1800460 (TPMT*3A, c.460G>A)  rs1142345 (TPMT*3A, c.719A>G)  rs1800460 (TPMT*3A, c.460G>A)  [rs1142345](https://www.ncbi.nlm.nih.gov/projects/SNP/snp_ref.cgi?rs=rs1142345) (TPMT*3C, c.719A>G) | - lower enzyme activity - more prone to bone marrow suppression - low TPMT activity: mutant homozygote - moderate TPMT activity: mutant   heterozygote   - high TPMT activity: no mutation | **Low TPMT activity:** avoid AZA or reduce daily doses by 10 times;  **Moderate TPMT activity:** initial dose reduction of 30%-80% or avoid AZA;  **High TPMT activity:** standard initial dose(Relling et al., 2019). |
|  | NUDT15 | NUDT15*1 (wild-type)；  rs746071566 (NUDT15*2)  rs116855232 (NUDT15*3) | - lower enzyme activity - more prone to bone marrow suppression - poor NUDT15 metabolism: without   the NUDT15*1 allele   - intermediate NUDT15 metabolism:   NUDT15*1-containing heterozygotes   - normal NUDT15 metabolism:   NUDT15*1 homozygote | **Poor NUDT15 metabolism:** avoid AZA or reduce daily doses by 10 times;  **Intermediate NUDT15 metabolism**: initial dose reduction of 30%-80%;  **Normal NUDT15 metabolism:** regular initial dose(Relling et al., 2019). |
|  | ITPA | [rs1127354](https://www.pharmgkb.org/variant/PA166155915) (ITPA, c.94C>A)  rs7270101 (ITPA, IVS2+21A>C) | - lower enzyme activity - increase in ADR |  |
| **CyA** | CYP3A4 | rs35599367 (CYP3A4*22, 522-191C>T) | - lower enzyme activity - reduce individual clearance - related to nephrotoxicity |  |
|  | CYP3A5 | rs776746 (CYP3A5*3, 6986A>G) | - decrease or loss of enzyme activity |  |
|  |  | CYP3A5*1 (wild-type) | - increases the risk of nephrotoxicity |  |
|  | ABC | rs717620 (ABCC2, c.-24C>T)  rs2231142 (ABCG2, c.421C>A) | - increase the risk of hepatorenal   toxicity |  |
| **TAC** | CYP3A5 | rs776746 (CYP3A5*3, 6986A>G) | - decrease or loss of enzyme activity - CYP3A5 non-expression type | For solid organ transplanters:  **CYP3A5 non-expression type**: start treatment according to the standard recommended dose (0.15mg/kg/d);  **CYP3A5 expression**: increase the initial dose by 1.5-2 times(Woillard et al., 2017). |
|  |  | CYP3A5*1 (wild-type) | - CYP3A5 expression type |  |

Abbreviations: CS=corticosteroids; NR3C1=nuclear receptor subfamily 3 group C member 1; CRHR1=corticotropin-releasing hormone receptor 1; GLCC1=glucocorticoid-induced transcript 1 gene; STIP1=stress-induced phosphoprotein 1; HDAC=histone deacetylase; ABC=ATP-binding cassette transporter; ONFH=osteonecrosis of the femoral head; PAI-1=plasminogen activator inhibitor-1; CYC=Cyclophosphamide; CYP=cytochrome P450; ADR=adverse reactions; GST=glutathione S-transferase; MMF=Mycophenolate mofetil; IMPDH=inosine monophosphate dehydrogenase; UGT=uridine diphosphate glucuronosyltransferase; AZA=azathioprine; TPMT=thiopurine methyltransferase; NUDT15=nucleoside diphosphate-linked moiety X-type motif 15; ITPA=inosine triphosphate pyrophosphatase; CyA=Cyclosporin A; TAC=tacrolimus.

**Reference:**

Relling, M.V., Schwab, M., Whirl-Carrillo, M., Suarez-Kurtz, G., Pui, C.-H., Stein, C.M., et al. (2019). Clinical Pharmacogenetics Implementation Consortium Guideline for Thiopurine Dosing Based on TPMT and NUDT15 Genotypes: 2018 Update. *Clinical Pharmacology & Therapeutics* 105(5)**,** 1095-1105. doi: 10.1002/cpt.1304.

Woillard, J.-B., Chouchana, L., Picard, N., Loriot, M.-A., and French Network, P. (2017). Pharmacogentics of immunosuppressants: State of the art and clinical implementation - recommendations from the French National Network of Pharmacogenetics (RNPGx). *Therapie* 72(2)**,** 285-299. doi: 10.1016/j.therap.2016.09.016.
